# Survey of Mast Cell Density in Transitional Cell Carcinoma

**DOI:** 10.30699/IJP.2020.123562.2345

**Published:** 2020-12-20

**Authors:** Hedieh Moradi Tabriz, Maedeh Obohat, Farzan Vahedifard, Arezoo Eftekharjavadi

**Affiliations:** 1 *Department of Pathology, Sina Hospital, Tehran University of Medical Sciences, Tehran, Iran*; 2 *Department of Neurology, Firoozgar Hospital, Iran University of Medical Sciences, Tehran, Iran *

**Keywords:** Mast cell tryptase, Transitional cell carcinoma, Urinary bladder, Mast cell density

## Abstract

**Background & Objective:**

Transitional cell carcinoma (TCC) is the world's seventh most common tumor and forms more than 90% of urinary bladder tumors. Invasive tumors are associated with poor prognosis, even with surgical treatment and chemotherapy. Some studies have found that an increase in the number of mast cells in TCC is related to the tumor grade and its aggressiveness. This study investigated the relationship between mast cell density (MCD) and features of TCC (tumor stage, grade, prognosis, and recurrence).

**Methods:**

Fifty-one cases with TCC were selected, and MCD was determined by immunohistochemistry (IHC) and Giemsa staining. Mortality rate and tumor recurrence were recorded.

**Results:**

The MCD mean was higher in high-grade tumors than in low-grade tumors (in IHC method: 9.127 vs 5.296; in Giemsa method: 5.512 vs 2.608). Also, the MCD mean in dead patients was higher than in survived patients (in IHC method: 11.390 vs 6.211; in Giemsa method: 7.460 vs 3.35). Patients with tumor recurrence showed a higher MCD mean than those without recurrence (in IHC method: 9.395 vs 5.475; in Giemsa method: 5.715 vs 2.931).

**Conclusion:**

Using mast cell tryptase and Giemsa, MCD may be associated with a positive correlation with tumor grade in TCC. Correlations between MCD, recurrence, prognosis, and tumor stage are probably caused by the effect of tumor grade (all with *P*<0.05).

## Introduction

Bladder cancer is the ninth most common cancer worldwide (1), and its incidence is growing. In Iran, this tumor in men ranked the fourth most common tumors (2). Transitional cell carcinoma (TCC) is the world's seventh most common tumor and forms 3.2% of all cancers and more than 90% of urinary bladder tumors (3, 4). The main determinants of prognosis for patients include tumor stage, lymph node involvement, histologic grade, age, tumor location, tumor size, and vascular invasion. Tumor stage is the most effective factor in the prognosis of these patients (5). Tumor invading muscularis propria significantly reduced survival rate (6). In Iran, the survival rate of patients with urinary bladder tumors is less than what has been reported elsewhere (7). 

Invasive tumors are associated with a poor prognosis, even with surgical treatment and chemotherapy based on tumor grade. That is why the analysis of new effective factors on the grade, stage, and prognosis of the tumor are of critical value in the treatment of the tumor, and mast cells would be one of these factors.

Mast cells originate in the bone marrow as CD34+ and CD117+ progenitors, migrate to the tissues by binding to integrin and vascular adhesion molecules, then develop into mature cells (8). There are two groups of mast cells in the human body:

- Connective tissue-like mast cells with expression of tryptase and chymase
- T mast cells containing tryptase

In some studies, their role in the development of new targeted anticancer therapies has been evaluated (9). 

Several surveys have suggested contradictory and different correlations (positive, negative, or even zero) of mast cells count with human tumors progression. A few studies have investigated the correlation between mast cell density (MCD) and tumor grade or stage, showing that higher density is accompanied by a high-grade tumor (10-13). In one study, the correlation has not been seen, but the density of these cells has been increased in tumor tissue compared to the surrounding normal tissue (14).

Since now, few studies have been done to examine the correlation between MCD and features of the tumor. Hence, we studied the relationship between MCD and tumor stage, grade, prognosis, and recurrence of TCC.

## Materials and Methods

The study was performed as a mixed cohort (including retrospective and prospective). First, pathology reports of patients with bladder cancers were reviewed from 2016 to 2018 recorded in the archive of the Department of Pathology in the Sina Hospital, Tehran, Iran. Cases with diagnosis of TCC (biopsied using the transurethral resection method and without the history of the previous diagnosis of TCC or treatment for this tumor) were selected (51 from 118 reviewed cases). 

Then, the hematoxylin and eosin-stained slides were blindly re-examined by an expert pathologist and re-classified based on the grade (consensus by the World Health Organization [WHO]/International Society of Urological Pathology [ISUP, 1998]) and stage of the tumor (tumor-node-metastasis [TNM] staging system proposed by the American Joint Committee on Cancer [AJCC] T2, T1, and Ta). Finally, an appropriate paraffin block of each sample (containing a sufficient amount of tumor, without necrosis and hemorrhage) were selected and cut into two sections of 3-micron thickness and separately stained by the immunohistochemical (IHC) markers for mast cells including tryptase and Giemsa stains.

For IHC staining, after deparaffinization in xylene and rehydration in a graded series of alcohol, the slides were rinsed with phosphate-buffered saline (PBS). Then, the activity of endogenous peroxidase was inhibited by hydrogen peroxide 3% for 30 minutes. After rinsing again with PBS, antigens were retrieved by placing the samples in Tris buffer 0.01 M, pH=9, in an autoclave at 120°C for 20 minutes. To reduce the nonspecific antibody binding, the slides were incubated with Protein Block Serum-Free (code X0909, Dako, Denmark) at room temperature for 10 minutes. The samples were incubated by (Monoclonal mouse anti-human, Clone 10D11, Novocastra) mast cell tryptase antibody (dilution: 1/80) for one hour.

Rinsing again with PBS, the slides were incubated with peroxidase-conjugated envision dual-link reagent (Rabbit/Mouse, Horseradish peroxidase, code K5007, DAKO, Denmark) for 30 minutes. Then, the samples were exposed to Aminobenzene Substrate for 10 minutes. The final step is to stain the background with hematoxylin. It should be noted that in each working set of IHC staining as the positive control, bone marrow biopsies from patients with aplastic anemia were used.

For Giemsa staining, the slides were placed in Giemsa solution (dilution: 1/10), a product from Bahar Afshan, for 10 minutes. Finally, the slides were rinsed with water (Figure 3).

After staining, each slide was blindly evaluated by an experienced pathologist, and three to five areas were selected with higher immunoreactivity (hot spots).

The number of stained cells (cytoplasmic pattern of staining) was counted in 10 high-power fields (HPFs) that were randomly chosen. The mast cells (MCs) were counted within tumor cells and stroma––according to similar studies (10, 14). 

**Fig. 1:**
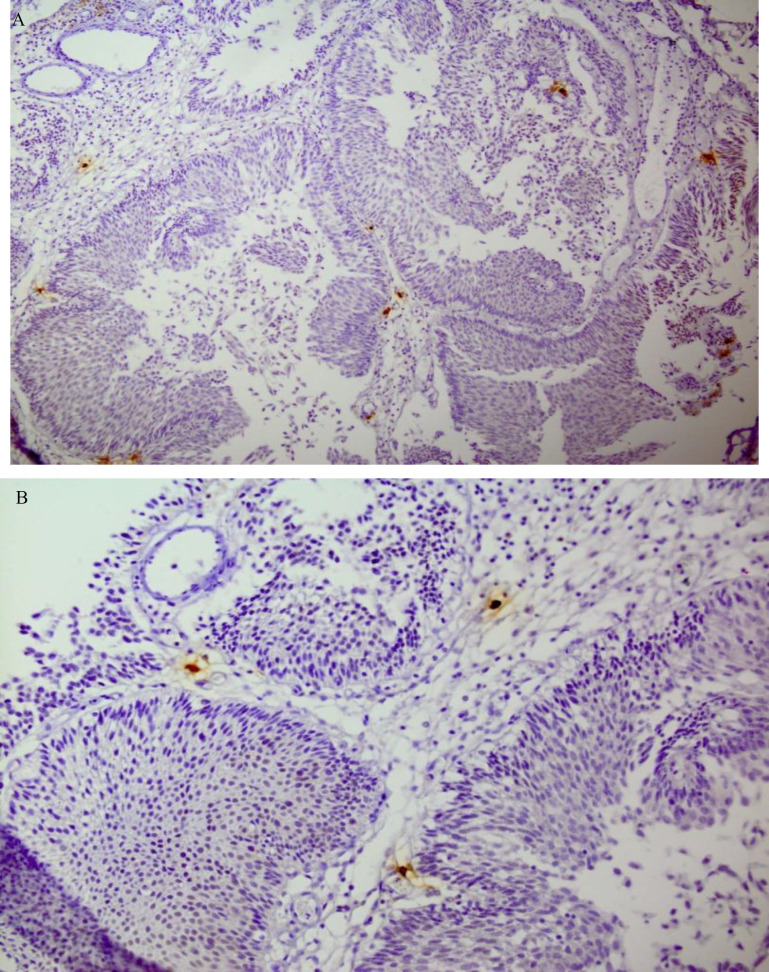
A and B: (X10, X20 magnification, respectively)

**Fig. 2:**
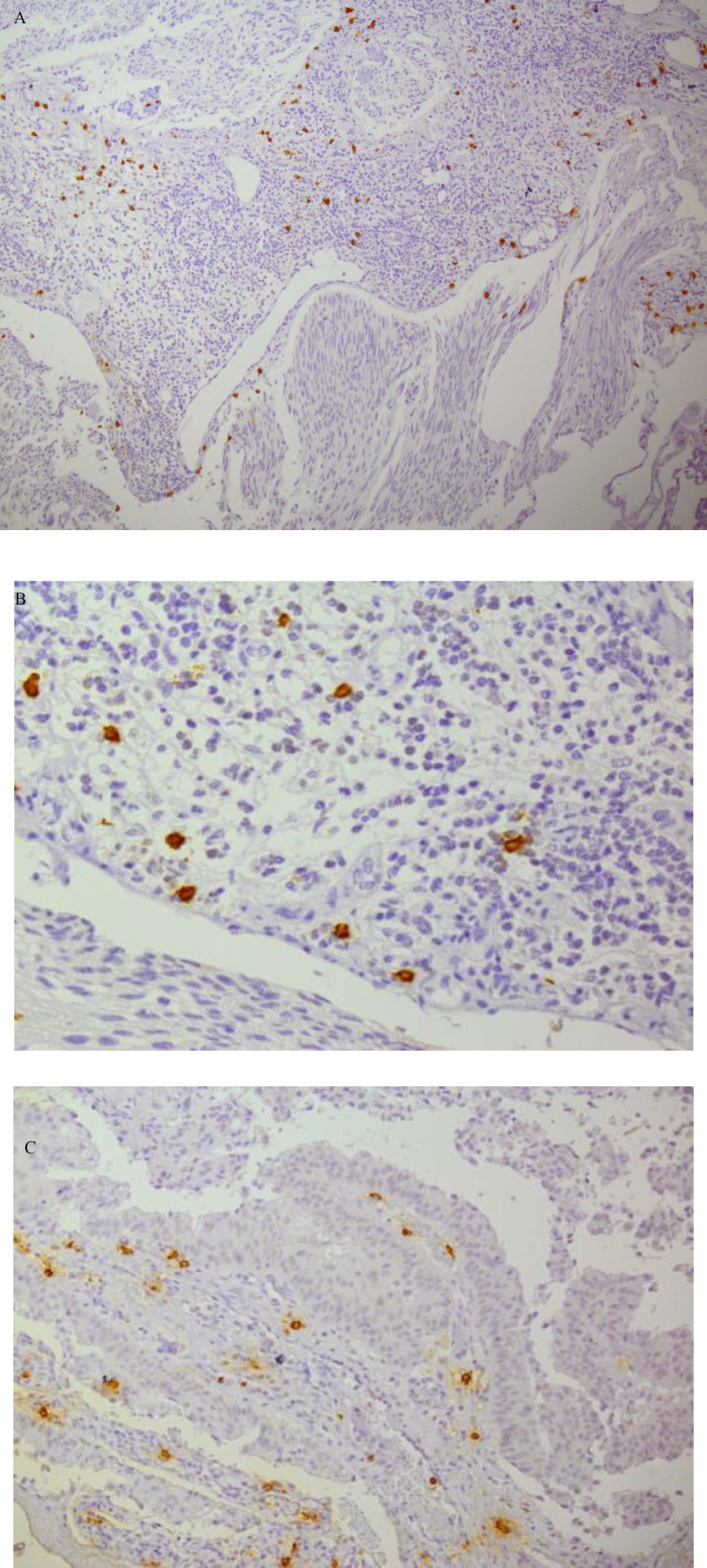
A, B, and C: (X10- X20- X40 magnification, respectively)

**Fig. 3:**
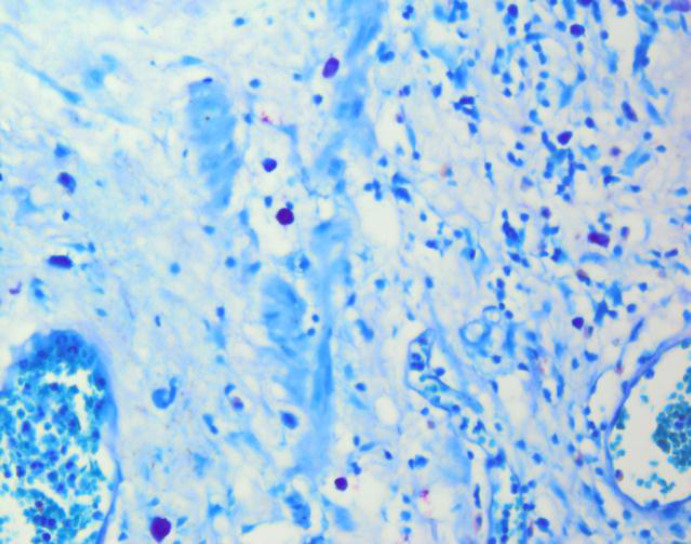
Giemsa staining for mast cells in TCC

The obtained numbers were averaged, which was reported as MCD per HPF (Figures 1 and 2 for mast cell tryptase, and Figure 3 for Giemsa staining).

Low-grade TCC (transitional cell carcinoma) stained by mast cell tryptase, highlighting mast cells

High-grade TCC stained by mast cell tryptase, highlighting mast cells

The follow-up of the patients was performed, and the patients were monitored for mortality rate and tumor recurrence. Mortality was defined as death caused by tumors or tumor complications. Recurrence was considered as the reoccurrence of tumors in the initial location of the tumor or metastasis to other organs after a period in which cancer has not been detected.

Finally, using SPSS 14 (SPSS Inc., Chicago, Ill., USA) and statistical tests, such as *t *test, paired *t *test, analysis of variance (ANOVA), Spearman, and Pearson correlation coefficient, the relationship of MCD (obtained from both methods of staining) with the grade, stage, prognosis, and recurrence were evaluated. P-value<0.05 was considered significant.

## Results

**Demographic Data**

Totally, 51 patients with the diagnosis of urinary bladder TCC without any history of previous treatment were evaluated. The sampling was done in such a way that the same ratio of the two levels of tumor grade was maintained relatively; thus, 26 samples were high-grade, and 25 samples were low-grade tumors. 

Another studied variable was tumor stage (Table 1), which was distributed relatively equal at three different levels, including Ta, T1, and T2 stages (respectively 19, 13, and 19). According to the usual distribution of TCC, low-grade tumors tend to be on the lower stages, and the high-grade ones tend to be on the higher stages. 

The mean and median of patients’ age were 67.36 and 69; retrospectively; 12% were female, and 88% were male. On follow up, ten patients died of tumor-related causes, and information about tumor recurrence in 36 individuals was obtained (20 patients presented with recurrence). The mean follow-up time was 18.92 months (ranges from 3 to 46 months).

**MCD in Different Methods**

The mean of MCD by IHC in high-grade tumors was 9. 127 and in low-grade samples was 5.296. There was a positive correlation between tumor grade and MCD using IHC (r=0.527; *P*=0.001 <0.05). According to the *t *test (*P*=0.013 <0.05) results, tumor grade was associated with MCD using IHC, and MCD in high-grade tumors was significantly higher than in low-grade.

The MCD mean by Giemsa staining in high-grade tumors was 5.512 and in the low-grade was 2.608. There was a positive correlation between tumor grade and MCD using Giemsa staining (r=0.541; *P*=0.001<0.05). Based on the *t *test results (*P*=0.001<0.05), the average of MCD in high-grade tumors was significantly higher than in low-grade tumors. Therefore, using two different stains, high-grade tumors presented with higher MCD compared to low-grade tumors. 

In the IHC method, there was a positive correlation between tumor stage and MCD (by r =0.541; *P*=0.001<0.05 and ANOVA with *P*=0.05). The least significant difference (LSD) analysis showed an only significant difference between the means of MCD in T2 stage with Ta, as MCD in tumors with T2 stage is greater than with Ta.

ByGiemsa method of staining, the correlation between tumor stage and MCD (by r =0. 448; *P*= 0 < 0.05; and ANOVA test with *P*=0.014 ≤ 0.05) was significant and positive. By using LSD, only the MCD mean of Ta and T2 showed a significant difference with each other, as MCD in tumors with stage T2 is greater than with Ta. 

A significant positive correlation was found between MCD using IHC and MCD using Giemsa staining (r=0.778, P=0.001). The paired *t *test revealed that MCD (sig=0) using IHC staining was significantly higher than using Giemsa staining.

A significant positive *P*=0.012<0.05) correlation was observed, and a significant difference was found between the mean MCD of the dead patient group and other groups. The results of the *t *test showed that the average of MCD in the first group was higher than in the second group (11.390 vs 6.211; *P*=0.022<0.05).

Although the positive relationship was seen between MCD (using IHC) and recurrence (r= 0.444, *P*=0.007 <0.05), according to the *t *test results (*P*=0.062 >0.05), the averages of MCD showed no difference between the group with recurrence and those without recurrence.

We found a positive correlation between MCD using Giemsa staining and the prognosis (r =0.516; *P*= 0.001 < 0.05). Furthermore, the mean of the mast cells count in the dead patients (7.460) were higher than alive subjects (3.356); using *t *test (*P*=0.002 <0.05). Also, MCD using Giemsa staining showed a positive relationship with recurrence. The results of the *t *test indicated that mean of the mast cells count was higher in the group with recurrence of the tumor (5.715), compared to the group without recurrence (2.931), (*P*=0.025<0.05), 

In low-grade tumors, the MCD mean using Giemsa staining showed no significant difference between the two groups of the patients with and without recurrence (Mann-Whitney test, *P*=0.086); the same conclusion was achieved for these two groups in high-grade tumors (*P*=1).

In low-grade tumors, the average of MCD using IHC was different between the dead patients and others (Mann-Whitney test, *P*=0.045); however, this difference was not notable in high-grade tumors (*P*=0.136).

In low-grade tumors, the averages of MCD between the group with Ta stage and with T1 stage were almost the same using both methods of staining (in IHC with *P*=0.617; in Giemsa with *P*=0.314). The same results yielded in high-grade tumors with stage T1 and T2 (in IHC with *P*=0.351; in Giemsa with *P*=0.434).

**Table 1:** *Crossover frequency table according to tumor grade and stage*

Total	Tumor stage			
	$T_{a}$	$T_{1}$	$T_{2}$	
26	**0**	**7**	**19**	**High-grade**
25	**19**	**6**	**0**	**Low-grade**
51	**19**	**13**	**19**	**Total**

## Discussion

Mast cells play an essential role in immune responses and allergic reactions. Several molecules related to tumor proliferation are produced by mast cells, including vascular endothelial growth factor (VEGF), angiopoietin, heparin, tumor necrosis factor α (TNF-α), fibroblast growth factor (FGF), interleukin 4 (IL-4), and so forth (15-18). Mast cells produce some critical molecules for tumor angiogenesis (19-21). They also contain heparin, which reduces the size and number of tumor colonies resulting in support for the hypothesis of mast cell antitumor effect (19).

Consequently, mast cells have a dual role and apposite function in human tumors, and the only way leading to tumor growth is the secretion of a selective substance without de-granulation (22). The state of mast cell differentiation and activation, as well as the local stromal environment, is essential for the type of molecule that is produced, stored, or secreted; this could be an explanation for their dual role (23, 24). The association between poor prognosis and presence of mast cells in rectal carcinomas, malignant melanomas, multiple myelomas, endometrial carcinomas, and Hodgkin lymphomas has been proven (25-30). However, the presence of mast cells in breast and ovarian cancer is associated with a better prognosis (31, 32).

The relationships between MCD in TCC and tumor stage and grade have been investigated in few studies but studies on prognostic significance of mast cells in TCC have not been fully performed yet. In our study, 51 patients with TCC were evaluated for investigation on relationships between MCD and tumor grade stage, recurrence, and prognosis. Information on each patient, including age, sex, density of mast cells (using IHC and Giemsa staining methods), tumor grade, stage, prognosis, and recurrence, were collected.

The sample size was calculated based on the grade variable, so that 26 out of the 51 samples were diagnosed as high-grade tumors, while 25 cases presented with low-grade tumors. The mean and median of patients’ age were 67.36 and 69 retrospectively, with the symmetric distribution.

In the case of the relationship between MCD and tumor grade, our results were similar to the study of Kim *et al.* (2011) (10), where MCD using IHC (mast cell tryptase) in high-grade tumors (134.9±47.99) was higher than in low-grade tumors (78.25±60.68; *P*<0.05).However, MCD in that study was differently evaluated compared to our study, since 5 high power fields X 200) were chosen by them. In that study, toluidine blue staining was the alternative method. Mast cell counts were significantly higher using IHC staining for tryptase compared with those obtained using toluidine blue staining.

The same results were also achieved in another study by Sari *et al.* (2011), as the means of MCD in low- and high-grade tumors were 5.3 and 14, respectively, with a significant difference. In that study, CD117 was used as an IHC marker (11).

In a survey by Serel *et al.* (2004), 56 samples were examined for MCD using toluidine blue staining (14). Tumor grade was divided into three levels: grade I, grade II, and grade III; the means of MCD in grade I, grade II, and grade III were 2.27, 3.96, and 3.75, respectively. The relationship between tumor grade and MCD was not significant and it was consistent with our results (the staining method and grading system were not similar to ours). They counted mast cells within tumor tissue and lamina propria in 5 random HPFs (X400), and the mean of each was calculated.

In the initial evaluation, MCD in both methods showed a significant difference between stage Ta and T2. Still, since all of Ta tumors are low-grade and all of T2 are high-grade, probably the results were due to the relationship between tumor grade and MCD. However, in the study by Sari *et al.*, MCD in tumors with stage Ta was higher than in tumors with other stages (11).

In the initial investigation, it was found that the MCD mean of the dead patient group using both staining methods was greater than that of the other groups; no significant difference was noted between the averages of MCD in high-grade tumors with and without recurrence, as in low-grade tumors by Giemsa. Thus, except for MCD by IHC method in low-grade tumors, the relationship between MCD and prognosis in other groups was a dependent value (due to the effects of grade on prognosis).

Initially, using Giemsa method of staining, a positive correlation was found between MCD and recurrence. Considering the groups of low-grade and high-grade tumors, no significant differences were observed between MCD in the group with recurrence and the one without recurrence (which eliminates the effect of tumor grade on the correlation between MCD and recurrence). In other words, the correlation between MCD by Giemsa and recurrence was not an independent relationship.

Our findings in this study showed that using mast cell tryptase and Giemsa staining methods, MCD showed a positive correlation with the tumor grade. Using immunohistochemical method, MCD was significantly higher than MCD using Giemsa staining. This proposed that MCD in TCC could not be accurately evaluated with Giemsa staining alone. 

Although there were positive correlations between MCD and recurrence, prognosis, and tumor stage (between Ta and T2), those could be explained through the effect of tumor grade. The exception was the positive correlation between MCD (using IHC staining) in low-grade TCC and worse prognosis. To get more reliable results, especially for the last three correlations, it is necessary to investigate a larger sample size.

## Conclusion

Using mast cell tryptase and Giemsa, MCD has a positive correlation with tumor grade in TCC. Correlations between MCD, recurrence, prognosis, and tumor stage are probably caused by the effect of tumor grade (all with *P*<0.05).

## Funding Sources

This study did not receive any specific grant from any companies, funding agencies in the public, commercial, or not-for-profit sectors. 
